# Mental Health and Well-being Measures for Mean Comparison and Screening in Adolescents: An Assessment of Unidimensionality and Sex and Age Measurement Invariance

**DOI:** 10.1177/10731911231158623

**Published:** 2023-03-02

**Authors:** Louise Black, Neil Humphrey, Margarita Panayiotou, Jose Marquez

**Affiliations:** 1 The University of Manchester, UK

**Keywords:** adolescence, well-being, mental health, measurement invariance, screening

## Abstract

Adolescence is a period of increased vulnerability for low well-being and mental health problems, particularly for girls and older adolescents. Accurate measurement via brief self-report is therefore vital to understanding prevalence, group trends, screening efforts, and response to intervention. We drew on data from the #BeeWell study (*N* = 37,149, aged 12–15) to consider whether sum-scoring, mean comparisons, and deployment for screening were likely to show bias for eight such measures. Evidence for unidimensionality, considering dynamic fit confirmatory factor models, exploratory graph analysis, and bifactor modeling, was found for five measures. Of these five, most showed a degree of non-invariance across sex and age likely incompatible with mean comparison. Effects on selection were minimal, except sensitivity was substantially lower in boys for the internalizing symptoms measure. Measure-specific insights are discussed, as are general issues highlighted by our analysis, such as item reversals and measurement invariance.

Accurate measurement of adolescent mental health and well-being is vital for several reasons. First, adolescence confers significant vulnerability to mental health difficulties and low well-being, as a developmental phase marked by considerable physical, social, psychological, and environmental change (Blakemore, 2019; Jones, 2013; Solmi et al., 2021). The worst outcomes are typically seen for girls and older adolescents (Campbell et al., 2021; NHS Digital, 2018). Second, recent evidence points to *increasing* prevalence of such difficulties among adolescents, particularly since the onset of the COVID-19 pandemic (Collishaw, 2015; Vizard et al., 2020). Third, concurrent and prospective child and adolescent mental health are important for quality of life, health, labor-market, and other outcomes (Goodman et al., 2015). Accurate measurement is therefore key to improving our understanding (e.g., trends and interventions), supporting those in need, and prevention efforts (Humphrey & Wigelsworth, 2016; Rutter & Pickles, 2016). To support these objectives, evidence to support use of sum scores and measurement equivalence between groups of interest (e.g., sex and age) is needed to ensure inferences are accurate.

Self-report measures are often used, particularly in large-scale research, and enable direct access to young people’s thoughts and experiences (Bentley et al., 2019; Deighton et al., 2014). These direct insights are likely crucial to improving our understanding of mental health in adolescence, given known heightened emotionality and social sensitivity during this period (Rapee et al., 2019). Some standards for age appropriateness for adolescent measures exist, and it is generally agreed adolescents possess the cognitive capacity to self-report (Bell, 2007; de Leeuw, 2011; Omrani et al., 2018). Nevertheless, psychometric fitness for purpose of self-report measures must be examined. Indeed, evidence is mounting that poor development practices may contribute to noisiness in adolescent mental health and well-being data (Bentley et al., 2019; Black et al., 2020; Black, Panayiotou, & Humphrey, 2022; Wolpert & Rutter, 2018). There is therefore a need to interrogate existing measures further from a psychometric standpoint to ensure these can be robustly used.

In addition, definitions of mental health and well-being are far from universally agreed (Humphrey, 2018), and the “jingle-jangle” fallacy prevails (Brookman-Byrne, 2020). Some studies use *well-being* interchangeably with *symptoms* or *mental health difficulties* (e.g., Fuhrmann et al., 2021; Orben & Przybylski, 2019), while others argue they are distinct constructs (Iasiello & Agteren, 2020). Furthermore, proposed domains within general mental health and well-being frameworks, including for example, hedonic, eudaimonic, and complete state models (Ryff et al., 2021; Westerhof & Keyes, 2010), are often conceptually similar (Alexandrova & Haybron, 2016; Black, Panayiotou, & Humphrey, 2022). For instance, hedonic/subjective well-being is defined as the combination of life satisfaction and affect (Diener et al., 2018), thus sharing content with internalizing symptoms (Alexandrova & Haybron, 2016). Eudaimonic/psychological well-being also has a somewhat diffuse definition and can include autonomy, environmental mastery, optimism, personal growth, positive relations with others, purpose in life, and self-acceptance (Ryff et al., 2021). Depending on its operationalization, it can therefore overlap with a host of domains and experiences and is poorly defined (Kashdan et al., 2008). Nevertheless, despite this theoretical broad range, there is some evidence different aspects of mental health and well-being can be highly related statistically (Black et al., 2019; Disabato et al., 2016).

Therefore, since general mental health and well-being appears mired in conceptual inconsistency and siloing (Black, 2022), there is a need to provide comparison and an overview of approaches within the field. Moreover, while symptom-based measurement is often prioritized to understand disease burden (Costello, 2015), positive well-being indicators are increasingly included in large studies (e.g., NHS Digital, 2018). Several authors have suggested the additional insights afforded by positive approaches, compared to measuring only symptoms, may support early identification of poor mental health outcomes (Bartels et al., 2013; Black et al., 2021; Greenspoon & Saklofske, 2001; Iasiello & Agteren, 2020). However, psychometric insight to support this, including into the comparability of outcomes, is typically missing (Bentley et al., 2019; Black, Panayiotou, & Humphrey, 2022). We, therefore, set out to address this issue in the current paper.

Given these issues, we adopt a broad, inclusive approach that reflects all the domains of mental health and well-being proposed by the young people who were consulted in the development of the #BeeWell survey, a major well-being project (#BeeWell Research Team, 2021; BeeWell Youth Steering Group Members, 2021). We prioritized including all #BeeWell well-being domains because there has been a historic tendency not to involve young people in the development of mental health and well-being measures, meaning face (and therefore also content) validity is often unclear (Black, Panayiotou, & Humphrey, 2022). Our approach, rather than, for instance, omitting potentially more proximal domains such as autonomy, sought to somewhat mitigate this problem and provide wide-ranging insights. This approach is also supported by evidence that even theoretically distinct domains can be strongly correlated, suggesting they measure similar or even equivalent experiences (Black et al., 2019; Disabato et al., 2016). The domains included map onto a range of theoretical domains, including hedonic (e.g., life satisfaction), eudaimonic (e.g., autonomy) and complete state (e.g., internalizing symptoms) models of well-being. Our approach also reflects work that has considered such domains together under broad approaches (i.e., non-disorder-specific) in systematic reviews of brief self-report measures (Bentley et al., 2019; Black, Panayiotou, & Humphrey, 2022; Deighton et al., 2014).

## Uses of and Issues in Adolescent Mental Health and Well-being Measures

Adolescent mental health and well-being measures are often deployed in two ways: using means such as in research to understand trends and response to intervention, or using cut scores or percentiles to estimate prevalence or for screening. Irrespective of application, these methods rely on the assumption that all items underpinning a score reflect a unidimensional construct. Basic evidence is needed to understand whether proposed scoring structures are empirically supported, and analysis should also be conducted to consider whether measures function similarly across groups (Flake et al., 2017). Where sum scores (observed unweighted totals) are used for group comparison, invariance of item intercepts, in particular, is important to infer valid mean comparisons (Steinmetz, 2013). For screening applications or prevalence reporting (which also often use cut-points, for example, Deighton et al., 2019), the impact of non-invariance (of loadings and intercepts) on selection should also be evaluated (Millsap & Kwok, 2004). Despite these clear guidelines, the current landscape appears to be poor, with robust evidence of dimensionality and invariance often particularly lacking (Bentley et al., 2019; Black, Panayiotou, & Humphrey, 2022).

Establishment of such properties is especially important for use outside research where further checks and nuanced decisions are unfeasible. However, researchers should ideally ensure planned analyses were appropriate for their data/questions, and check/accommodate underpinning measurement assumptions (Flake & Fried, 2020). Adolescent mental health and well-being measurement efforts, however, are often focused in schools, which are increasingly viewed as an appropriate setting to gather data for assessment/monitoring and/or screening purposes (Humphrey & Wigelsworth, 2016). Although this provides significant opportunities, measures in such settings are typically analyzed via simple sum scores. Crucially, while complex modeling (e.g., structural equation models, including partial invariance) may help clarify how measures should ideally be used, and help accommodate issues in research, this cannot be applied in schools. There is, therefore, a case that for measures being used in schools, particularly high psychometric standards should be met. Reliance on sum scores and often arbitrary cut points where these are not justified, risks missing those in need, and misunderstanding intervention response or trends. Although we, therefore, stress that there are particular risks associated with deployment in schools, psychometric work nevertheless has direct implications for research where measurement assumptions are frequently underexamined (Flake et al., 2017; Flake & Fried, 2020).

Consistent with this generally poor psychometric landscape, the quality and quantity of the underpinning evidence base for school applications remain limited (Soneson et al., 2020). Use of bespoke, unvalidated measures is also the norm (NatCen Social Research & National Children’s Bureau, 2017). Importantly, the intended purpose of data gathering can have implications for measure choice (Patalay & Fried, 2020). For example, assessment and monitoring may lead to briefer measures being favored (as they are typically delivered as part of a battery) than when screening (where longer measures may be preferable; Rammstedt & Beierlein, 2014; Ziegler et al., 2014). Clearly, therefore, there is a need to provide insight into which measures are most suited to simple sum scoring, use for mean comparison, and for selection.

To support implications for schools we focus specifically on age and sex equivalence for two substantive reasons: First, these inequalities are typically the most marked (Campbell et al., 2021; Casas & González-Carrasco, 2019; The Children’s Society, 2021; NHS Digital, 2018), and therefore frequently of interest. Second, the distribution of sex and age will typically be similar across school settings, likely making our findings more generalizable. For pragmatic reasons (i.e., availability in the dataset used), we focus specifically on the differences between ages 12 and 13 versus 14 and 15. However, this phase of development also represents a period marked by sharp increases in problems (Rapee et al., 2019), as well as changes in cognition and reading ability relevant to questionnaire responding (de Leeuw, 2011). Both of these issues are in turn highly relevant to measurement invariance.

Beyond scoring and sex/age comparisons, there is a need to understand the empirical similarity of measures given the fact different domains are sometimes used interchangeably (e.g., Fuhrmann et al., 2021; Orben & Przybylski, 2019), or additively (Iasiello & Agteren, 2020). Insight is therefore needed to inform how likely results are to vary depending on measure/domain operationalization (Carlson & Herdman, 2012). Such convergent validity evidence would also provide a necessary (but not sufficient) condition for construct validity (Franke et al., 2021). This convergence information, in combination with insight into dimensionality and invariance, could also aid in decisions about which measures to choose (e.g., a set of measures that are unique but unbiased versus congruent but biased).

## The Current Study

In this paper, we draw on a unique contemporary dataset (#BeeWell Research Team, 2021) that contains data on a range of multi-item measures spanning multiple candidate well-being domains (autonomy, optimism, general well-being, self-esteem, stress, emotion regulation, positive affect, and internalizing symptoms) for nearly 38,000 adolescents aged 12 to 15. These data were used to assess (uni)dimensionality via a range of factor analytic and network psychometric methods, thus providing insight into their appropriateness for sum-scoring. We also considered measurement invariance across sex and age to determine the impact of any non-equivalence on mean comparison and selection. Finally, we considered the convergence of measures to provide insight into the potential impact of selecting a given measure/outcome on results. Collectively, our analyses aim to provide thorough insight into some of the most fundamental measurement issues that ought to underpin prevalence and screening efforts. Such insights are important given the proliferation of these kinds of measures, their increasing use for school-based assessment, monitoring and screening purposes, and the relative lack of rigorous underpinning psychometric evidence.

## Method

### Sample

The #BeeWell time one sample consisted of 37,978 adolescents from 165 schools (99.29% attended mainstream though a small proportion of special schools and alternative provision were included). We excluded those participants who had missing data for all survey variables included in this study, resulting in a sample of 37,149 who responded to at least one item considered here. Of this sample, 49.34% were female and 50.66% were male, 53.63% were in year 8 (aged 12–13) and 46.37% were in year 10 (aged 14–15). 24.72% had been eligible for free school meals in the last 6 years, and 13.80% were identified as having special educational needs. In terms of ethnicity, 17.73% were from Asian backgrounds, 5.24% were Black, .78% Chinese, 5.68% Mixed, 1.83% unclassified, 64.60% White, and 2.22% were from any other ethnic background (1.92% had missing ethnicity data). Overall these results are mostly similar (within a few percentage points) to national averages for England, though the exact free-school meal metric is not comparable, and the current sample had higher rates of Asian students than national figures (Gov.uk, 2022).

### Measures

Measures in the #BeeWell study were selected through an extensive consultation process. This involved more than 150 young people in workshops designed to facilitate an understanding of what well-being means to them, and the factors that influence their well-being. These workshops were combined with inputs from an expert multi-stakeholder advisory group (e.g., academic researchers, mental health professionals, health care representatives, education experts, parents) to inform the domains covered in the survey. The #BeeWell research team sought established (i.e., some documented research development history), non-proprietary self-report measures, bringing options to the advisory group, and seeking their feedback alongside that of young people (#BeeWell Research Team, 2021). The multiplicity of perspectives meant that the final selection of measures was informed by a range of issues, including (but not limited to) face validity, psychometric evidence, completion burden, accessibility, and meaningfulness. The wording of all items used in the current paper is available at https://gmbeewell.org/wp-content/uploads/2021/09/BeeWell-Questionnaires-Booklet.pdf where the wider survey can be viewed as a whole.

Table 1 provides a basic overview of the measures.

**Table 1.** Overview of Measures.

Domain	Measure	Subscale used and/or version notes	*N* of items	Sample item	Response format	Scoring and interpretation
Optimism	Engagement perseverance optimism connectedness happiness (EPOCH-O)	Optimism subscale (Kern et al., 2016)	4	I am optimistic about my future	Almost never, sometimes, often, very often, always	Sum score, higher score = greater optimism
Autonomy	Basic Psychological Need Satisfaction and Frustration Scale (BPNSFS-A)	Autonomy subscale (Deci & Ryan, 2000)	6	I have enough choice about how I spend my time	1 = completely not true to 5 = completely true	Sum score, higher score = greater autonomy; 2 items reverse scored
Mental well-being	Short Warwick Edinburgh Mental Well-being Scale (SWEMWBS)	Stewart-Brown et al. (2009)	7	I’ve been thinking clearly	None of the time, rarely, some of the time, often, all of the time	Sum score, higher score = greater mental well-being
Self-esteem	Rosenberg Self-Esteem Scale (RSS)	5-item short version for adolescents deployed in Millennium Cohort Study (Schoon et al., 2015)	5	I feel good about myself	Strongly agree, agree, disagree, strongly disagree	Sum score, higher score = higher self-esteem
Stress	Perceived Stress Scale (PSS-4)	4-item short version (Cohen et al., 1983)	4	In the last month, how often have you felt that you were unable to control the important things in your life?	Never, almost never, sometimes, fairly often, very often	Sum score, higher score = higher stress; 2 items reverse scored
Emotion regulation	Trait Emotional Intelligence Questionnaire Adolescent Short Form (TEIQue-ASF-ER)	Emotion regulation subscale (Petrides et al., 2006)	6	I can control my anger when I want to	1 = completely disagree to 7 = completely agree	Sum score, higher score = higher emotion regulation; 3 items reverse scored
Internalizing symptoms	Me and My Feelings scale (M&MF-I)	Emotional difficulties subscale; measure also referred to as Me and My School (Deighton et al., 2013)	10	I worry a lot	Never, sometimes, always	Sum score, higher score = higher internalizing symptoms; scores can be organized into three bands (normal, borderline, clinically significant)
Positive affect	Positive and Negative Affect Schedule: Child Version (PANAS-C-PA)	Positive affect subscale (Ebesutani et al., 2012)	5	Indicate to what extent you have felt happy during the past few weeks	Very slightly or not at all, a little, moderately, quite a bit, extremely	Sum score, higher score = greater positive affect
Life satisfaction	Office for National Statistics (ONS) Life Satisfaction item (Office for National Statistics [ONS], 2018)	Taken from ONS4 personal well-being item bank	1	Overall, how satisfied are you with your life nowadays?	0 = not at all to 10 = completely	Higher score = greater life satisfaction

*Note.* Life satisfaction item included in convergent validity analysis only.

### Statistical Analysis

All analyses were conducted in R^1^ with code, including packages used, provided at https://osf.io/zqfxb/. In addition, we also provide here synthetic data (the full dataset cannot be made publicly available until the end of the project since this would be anonymized and participants can withdraw their data until this time).

#### Dimensionality Assessment

We approached the assessment of unidimensionality in three stages. First, we fitted one-factor confirmatory factor analysis (CFA) models to determine if a measure’s intended unidimensionality was supported. Second, where CFA results were not acceptable (see below), we conducted exploratory graph analysis (EGA). Third, where multidimensionality was suggested by EGA, we estimated a bifactor model to consider the extent of multidimensionality via additional indices. Each step is explained in more detail in the subsequent sections.

##### Confirmatory Factor Analysis

We judged the fit of a unidimensional model for each measure using dynamic fit indices (DFI; McNeish & Wolf, 2021). This method can be applied with maximum likelihood (ML) estimation and has several advantages over canonical fit thresholds (Hu & Bentler, 1999). First, fit indices are sensitive to issues such as factor reliability and the number of items/factors, meaning that one set of cut-offs cannot generalize across modeling scenarios (McNeish & Wolf, 2021). In addition, unidimensional models should be judged against different types of misspecification to multidimensional models (e.g., error covariance rather than cross-loadings), making bespoke approaches all the more necessary (McNeish & Wolf, 2022).

The DFI method is a simulation-based approach that focuses on potential misspecification of a given model. For unidimensional models, as considered here, the standardized loadings and sample size are used for a series of simulations to determine the sensitivity of the comparative fit index (CFI), root mean square error of approximation (RMSEA), and standardized root mean square residual (SRMR) to four levels of misspecification. These are Level 0 (L0), no misspecification; Level 1 (L1), equivalent to residual covariance = .30 in a third of the items; Level 2 (L2), equivalent to residual covariance = .30 in two thirds of the items; and, Level 3 (L3), equivalent to residual covariance = .30 among all items. For measures with less than six items, the full range of misspecification levels cannot be calculated since there are not enough degrees of freedom to introduce all the necessary error covariances. Therefore, for 5-item measures (Rosenberg Self-Esteem Scale [RSS] and Positive and Negative Affect Schedule: Child Version [PANAS-C-PA]), only the first two levels were available, and for 4-item measures (Engagement perseverance optimism connectedness happiness [EPOCH-O] and Perceived Stress Scale [PSS-4]), only one level. Since the aim was to identify the most appropriate measures for use with simple sum scores, we only considered CFA evidence to point to unidimensionality if L0 was achieved across all three indices. Where this strict criterion was not met, we conducted additional analyses (EGA and bifactor indices, see below) to consider the extent of multidimensionality.

All measures except M&MF internalizing (Me and My Feelings scale [M&MF-I]) had five response categories or more, and did not exhibit substantial non-normality (see “Results” section), suggesting ML estimation, treating items as continuous, was appropriate (Rhemtulla et al., 2012). Since methods to treat items as ordinal are currently not available within the DFI framework, we also estimated the CFA for M&MF-I using the diagonally weighted least squares (DWLS) estimator to account for its three-point response format. Fit of the DWLS model was judged in line with widely-used cut-offs, CFI > .95, RMSEA < .06, SRMR < .08 (Hu & Bentler, 1999), since the DFI method cannot currently be applied with DWLS.

##### Exploratory Graph Analysis

Where measures did not satisfy unidimensionality according to CFA, we conducted EGA (Golino & Epskamp, 2017). This has been shown to perform well at identifying the number of dimensions items measure, and is particularly favorable where multiple dimensions are highly correlated (Golino et al., 2020), as was expected here given that measures were intended to be unidimensional. To match the analytical conditions in this simulation work, we used the default arguments for EGA, including the *cor_auto* function, meaning that polychoric correlations were used. Where EGA found only one factor, we considered this as evidence in favor of unidimensionality, and where multiple factors were found, this was used to inform bifactor modeling.

##### Bifactor Modeling

Where measures did not meet criteria for unidimensionality in CFA models, and evidence of multiple factors was found via EGA, we estimated bifactor models. Consistent with our CFA analyses, we used ML estimation for all measures except M&MF-I. Although bifactor models can be difficult to interpret (Eid et al., 2017), and subject to fit bias (Greene et al., 2019), they can be useful when considering the extent of unidimensionality via supplementary indices (Reise, Scheines, et al., 2013; Rodriguez et al., 2016). We, therefore, used the explained common variance (ECV) and percentage of uncontaminated correlations (PUC). These represent the percentage of variance explained by a general factor, and the ratio of observed data correlations that inform general versus specific factors, respectively. Where ECV and PUC are both > .70, measures can be considered essentially unidimensional since relative bias is likely to be slight (Rodriguez et al., 2016). Where measures met these thresholds, we, therefore, considered this evidence in favor of unidimensionality.

##### Judging Unidimensionality

Given the complexity of our unidimensionality assessment, we provide a summary of how results were integrated here. If DFI L0 was met across all indices, no further testing was required, and the measure was judged to be unidimensional. If mixed or limited support was seen for DFI, EGA was conducted. Where this showed only one factor, no further analysis was conducted, and the measure was considered to be unidimensional (assuming dramatic misfit, e.g., L3 misspecification for DFI was not seen in CFA). Where EGA showed more than one factor, bifactor indices were used to consider whether essential unidimensionality was supported. If essential unidimensionality was supported, we considered the measure to show sufficient evidence to be considered unidimensional. Our approach was therefore sequential, with the strictest CFA/DFI test conducted first.

#### Reliability

We estimated Cronbach’s alpha and McDonald’s composite ω reliability coefficients to assess internal consistency (assuming a unidimensional model). Although α assumes tau-equivalence (equal loadings for all items), and is therefore often a lower bound, ω is based on a unidimensional factor model, meaning item loadings can vary which is typically more realistic (McNeish, 2018). Reliability was not used to provide insight into unidimensionality, but was assessed to provide additional insight into total scoring. For instance, similar and high α and ω suggest observed sum scores may be supported (Widaman & Revelle, 2022). However, either coefficient might be undermined where assumptions such as local independence are clearly violated. Therefore, where unidimensionality was clearly not supported, reliability results were treated with caution.

#### Measurement Invariance Analysis

For measures to be comparable across groups, factor loading and intercept parameters should be consistent across groups. If a given measure was found to be unidimensional, we evaluated whether measurement invariance held across sex and age. Where clear evidence against unidimensionality was found, further invariance analysis was considered inappropriate.

##### Identification of Non-Invariant Parameters

First, baseline models in each group within sex and age (e.g., boys and girls separately) were estimated, and new DFIs were simulated to account for the change in sample size. Next, two measurement invariance models were estimated for each unidimensional measure in the total sample: configural, in which parameters were freely estimated in each group, and scalar, in which loading and intercept parameters were constrained to equality across groups. Metric invariance (equality constraints on loadings only) was not used at this stage to identify non-invariant loadings. This was for simplicity, and to avoid the problems associated with traditional invariance testing, that is, capitalizing on chance via modification indices (Luong & Flake, 2021). However, our subsequent alignment analysis to identify non-invariant parameters considered intercepts *and* loadings. Both non-invariant intercepts and loadings were, therefore, used to inform partial models for invariance testing.

Configural and scalar models were, therefore, compared to consider whether scalar invariance held, not to identify non-invariant parameters. This was done only through the automated alignment procedure (see below). Various methods to judge the result of this comparison are available: chi-square difference testing, CFI difference cut-off = .002 (Meade et al., 2008), and Akaike information criterion (AIC)/Bayesian information criterion (BIC) can be compared with the lower number in both cases indicating the preferable model (van de Schoot et al., 2012). Since each of these methods are sensitive to different issues such as factor reliability (Kang et al., 2016) and sample size (Crede & Harms, 2019), we report them all for transparency rather than relying on a single cut-off from any one. In addition, we expected full scalar invariance to be too strict (Luong & Flake, 2021), and therefore followed this with alignment analysis.

The alignment method optimizes an approximately invariant solution based on the fit of the configural model (Asparouhov & Muthén, 2014). This method automates the invariance testing process, rather than relying on modification indices and many decisions from researchers (Luong & Flake, 2021). Where full scalar invariance is not met, this method allows for consideration of measures with approximate measurement invariance, and allows non-invariant items to be identified. Since we compared groups with only two categories, we used alignment with fixed optimization (Luong & Flake, 2021). Although only ML is available for the DFI approach used to consider dimensionality, for alignment models we opted to use robust maximum likelihood (MLR) as an additional safeguard against non-normality. It is recommended that for group mean comparison with sum scores, intercept parameters should be invariant (Steinmetz, 2013). We, therefore, considered measures that met this criterion to be suitable for mean comparison across a given group.

##### Selection Bias

Measures can also be used to identify individuals above a threshold, either for further assessment, treatment, or to estimate prevalence. Using the method set out by Millsap and Kwok (2004), and elaborated by Lai et al. (2017), we estimated the effect of measurement non-invariance on selection across groups. Results of a partially invariant model (using the MLR estimator), including any non-invariant parameters identified in alignment analysis, were compared to sum scores to determine the bias of using a sum score to select those with the lowest well-being. The method provided by Lai et al. (2017) automates the calculation of a cut point based on a specified selection proportion in the total sample. This method was appropriate here since only M&MF-I and Short Warwick Edinburgh Mental Well-being Scale (SWEMWBS) have published cut points (Patalay et al., 2014), and in the case of SWEMWBS, these were not derived through analysis of adolescent samples (Ng Fat et al., 2017; Shah et al., 2021). Given the absence of appropriate cut points for all but one measure, and to apply a consistent approach, we used the default 25% selection proportion in all cases. We report the cut point, sensitivity, specificity and proportion selected for each group. We considered sensitivity and specificity to be minimally acceptable for screening at .70, consistent with thresholds for clinical validity (Sheldrick et al., 2015). For positive well-being (i.e., all measures except M&MF-I), all items were coded so that selection could be assessed in terms of those with lowest well-being, consistent with screening efforts.

#### Convergent Validity

For measures that were deemed to be unidimensional, we also estimated Pearson correlations between sum scores, as well as to the single life satisfaction item (see Table 1). This analysis allowed insight into the equivalence of each (e.g., as an outcome in a trial), since, as discussed above, different domains of mental health and well-being are sometimes used interchangeable or additively. Carlson and Herdman (2012) recommend that a threshold of *r* > .70 be used for convergent validity, since below this the difference in results between studies using different proxies was above *r* = .10 in 30% of cases.

## Results

### Descriptive Statistics

Missing data at the individual item level ranged from .07 to .09%, and for sex was < .01%. There were no missing data for year group. Skewness for individual items ranged in absolute value from .01-.93. These results, therefore, support the estimation procedures outlined above (Rhemtulla et al., 2012).

### Dimensionality Assessment

An overview of dimensionality results and reliability can be seen in Table 2. For ease of interpretation, we provide the level of misspecification based on DFI (where applicable), and our judgment as per the criteria described above, rather than all empirical fit indices. Empirical fit and DFI cut-off values, including bifactor models where applicable, can be found in supplementary Tables S1 and S2. The EPOCH optimism (EPOCH-O) measure and SWEMWBS showed no misspecification and were therefore not subjected to further EGA or bifactor analysis. The PANAS-C PA scale showed mixed results across CFI, RMSEA, and SRMR. M&MF-I was similarly borderline (meeting traditional CFI and SRMR but not RMSEA cut-offs) in terms of CFA (DWLS model). Both these measures were, however, determined to be unidimensional according to EGA. Similarly, the RSS showed L1 misspecification in terms of DFI but was unidimensional according to EGA. These five measures were, therefore, considered to be broadly supported as unidimensional and were taken forward for further analysis.

**Table 2.** Overview of Dimensionality Assessment Results.

Measure	CFA	EGA	Bifactor indices	Unidimensionality conclusion	Reliability
BPNSFS-A	L1	Two factors	ECV=.65, PUC=.53	−	α = .71 ω = .74
TEIQue-ASF-ER	L3	Two factors	ECV=.45, PUC=.60^a^	−	α = .63 ω = .64
M&MF-I	DCFA: L2/L3 DWLS: CFI=.985, RMSEA=.095, SRMR=.06	Unidimensional	NA	+	α = .88 ω = .88
EPOCH-O	L0	Unidimensional	NA	+	α = .81 ω = .81
PANAS-C-PA	L0/L1	Unidimensional	NA	+	α = .92 ω = .92
SWEMWBS	L0	Unidimensional	NA	+	α = .86 ω = .86
RSS	L1	Unidimensional	NA	+	α = .91 ω = .91
PSS-4^b^	ML fit: CFI=.571, RMSEA=.321, SRMR=.157^c^	Two factors	ECV=.24, PUC=.67	−	α = .57 ω = .61

*Note.* CFA = confirmatory factor analysis; EGA = exploratory graph analysis; BPNSFS-A = Basic Psychological Need Satisfaction and Frustration Scale; ECV = explained common variance; PUC = percentage of uncontaminated correlations; M&MF-I = Me and My Feelings scale; DWLS = diagonally weighted least squares; DCFA = dynamic confirmatory factor anlaysis; CFI = comparative fit index; RMSEA = root mean square error of approximation; SRMR = standardized root mean square residual; TEIQue-ASF-ER = Trait Emotional Intelligence Questionnaire Adolescent Short Form; EPOCH-O = Engagement Perseverance Optimism Connectedness Happiness; PANAS-C-PA = PANAS-C positive affect subscale; SWEMWBS = Short Warwick Edinburgh Mental Well-being Scale; RSS = Rosenberg Self-Esteem Scale; PSS-4 = Perceived Stress Scale; L0 = no misspecification; L1 = Level-one misspecification; L2 = Level-two misspecification; L3 = Level-three misspecification.

a A six-item bifactor model with three items per specific factor is not identified, so one lambda estimate from an unidentified run was used to identify the model as recommended (Muthén & Muthén, 2021). ^b^ For both the unidimensional and bifactor PSS-4 models, a Heywood case (negative residual variance) was found for the second item. This was fixed to 0. ^c^ Serious problems with the unidimensional model for PSS-4 meant further testing was not appropriate or possible.

In contrast, the BPNSFS autonomy (BPNSFS-A), emotion regulation (Trait Emotional Intelligence Questionnaire Adolescent Short Form [TEIQue-ASF-ER]), and PSS-4 measures all showed substantial problems in the CFA models, two factors in EGA, and lacked essential unidimensionality according to bifactor indices. Factors suggested by EGA, and used in bifactor models, all related to positive/negative wording: For BPNSFS-A, the items about feeling pressure and having to do “what I’m told” were grouped separately from the remaining positively-framed items (e.g., “I feel like I am free to decide for myself how to live my life”); for TEIQue-ASF-ER the positively-framed items such as “I am able to deal with stress” were grouped separately from the negatively-framed items such as “I find it hard to control my feelings”; for PSS-4 the two stress items about being “unable to control” stress and “difficulties. . . piling up” were separate from the two coping items about “confident about your ability to handle your personal problems” and feeling that “that things were going your way.”

### Measurement Invariance

Though model fit did not lead to the same conclusions across ML and DWLS estimators for M&MF-I for the total sample (single-group) model, factor loadings (i.e., comparable parameters) were highly correlated, *r* = .97. Since we predominantly used fit as an optimization problem, rather than for difference testing, and given this similarity for parameter estimates, we opted to treat M&MF-I items as continuous for invariance testing. This enabled us to use a consistent factor analytic (rather than item response theory) framework when considering selection bias, given that analytical methods for selection bias with polytomous items are not available (Gonzalez & Pelham, 2020). In addition, as noted above, we were able to use MLR estimation for alignment and partially-invariant models as an additional safeguard.

#### Identification of Non-Invariant Parameters

Baseline models for each group (male vs female and Year 8 vs Year 10) for each measure tended to fit no worse than L1 misspecification for any given fit index which we deemed sufficient to proceed to the configural model (see supplementary Table S3). Exceptions to this were baseline models for RSS in the Year 10 group and for M&MF-I models. However, configural models were all deemed acceptable (see supplementary Table S4). Of the five measures taken forward for measurement invariance analysis, none clearly achieved scalar invariance: A significant difference in model fit was found between configural and scalar models in all cases; The difference between CFI for pairs of configural/scalar models ranged from .001 to .035; AIC was consistently *worse* for scalar models and BIC similarly favored the configural model in 8 out of 10 cases (see supplementary Table S4). This behavior of BIC could be consistent with a known tendency to over favor more parsimonious models (Vrieze, 2012). Based on the balance of these results, we concluded scalar invariance was not supported for any measure. Although a few RMSEA values exceeded canonical fit cut-offs for configural/scalar models, when compared to the DFIs generated for the whole sample models (see supplementary Table S1), these were L1 or better.

We, therefore, proceeded to alignment testing for each of the five measures across both groups. Alignment results indicated a high proportion of non-invariant parameters (see Table 3 and supplementary Table S5), and therefore that mean comparisons for any of the five unidimensional models across sex and year group should likely be treated with caution. Fit of the partially-invariant models estimated based on the results of alignment analyses, and to inform selection bias testing can be found in supplementary Table S6.

**Table 3.** Percentage of Non-Invariant Parameters.

Measure/group	% noninvariant loadings	% noninvariant intercepts
EPOCH-O/sex	50	25
EPOCH-O/year	0	25
SWEMWBS/sex	71.43	85.71
SWEMWBS/year	14.29	71.43
RSS/sex	80	80
RSS/year	0	60
M&MF-I/sex	50	100
M&MF-I/year	30	70
PANAS-C-PA/sex	40	100
PANAS-C-PA/year	20	60

*Note.* EPOCH-O = Engagement Perseverance Optimism Connectedness Happiness; SWEMWBS = Short Warwick Edinburgh Mental Well-being Scale; RSS = Rosenberg Self-Esteem Scale; M&MF-I = Me and My Feelings scale; PANAS-C-PA = PANAS-C positive affect subscale.

##### Selection Bias

Results of selection bias analyses are shown in Tables 4 and 5. These show that sensitivity and specificity were typically similar across groups and acceptable (> .70), except for M&MF-I, where sensitivity was much lower for boys (.51), compared to .94 for girls.

**Table 4.** Selection Bias Results for Sex.

Measure	Cut score		Proportion selected		Sensitivity		Specificity	
	Girls	Boys	Girls	Boys	Girls	Boys	Girls	Boys
EPOCH-O	14.81	14.81	.31	.19	.78	.76	.91	.93
SWEMWBS	22.84	22.84	.33	.17	.82	.79	.92	.94
RSS	16.81	16.81	.18	.32	.82	.86	.96	.94
PANAS-C-PA	16.89	16.89	.31	.19	.93	.72	.90	.98
M&MF-I	9.86	9.86	.37	.13	.94	.51	.83	.99

*Note.* EPOCH-O = Engagement Perseverance Optimism Connectedness Happiness; SWEMWBS = Short Warwick Edinburgh Mental Well-being Scale; RSS = Rosenberg Self-Esteem Scale; PANAS-C-PA = PANAS-C positive affect subscale; M&MF-I = Me and My Feelings scale.

**Table 5.** Selection Bias Results for Age.

Measure	Cut score		Proportion selected		Sensitivity		Specificity	
	10	8	10	8	10	8	10	8
EPOCH-O	14.82	14.82	.27	.23	.78	.77	.92	.92
SWEMWBS	22.87	22.87	.27	.23	.82	.79	.93	.94
RSS	16.79	16.79	.22	.28	.84	.85	.95	.94
PANAS-C-PA	16.92	16.92	.28	.22	.85	.85	.95	.95
M&MF-I	9.9	9.9	.26	.24	.83	.82	.94	.94

*Note.* EPOCH-O = Engagement Perseverance Optimism Connectedness Happiness; SWEMWBS = Short Warwick Edinburgh Mental Well-being Scale; RSS = Rosenberg Self-Esteem Scale; PANAS-C-PA = PANAS-C positive affect subscale; M&MF-I = Me and My Feelings scale;10 = year 10; 8 = year 8.

### Convergent Validity

Correlations between unidimensional measure sum scores can be seen in Table 6. All were below the recommended minimum of *r* = .70.

**Table 6.** Total Score Means, Standard Deviations, and Correlations With Confidence Intervals.

Variable	*M*	*SD*	1	2	3	4	5
1. Life satisfaction	6.63	2.50					
2. EPOCH-O	11.76	3.80	.58** [.58, .59]				
3. SWEMWBS	23.08	5.82	.66** [.65, .66]	.66** [.65, .66]			
4. RSS	14.48	3.45	.62** [.61, .62]	.58** [.57, .58]	.64** [.63, .64]		
5. M&MF-I	6.71	4.73	−.61** [−.62, −.61]	−.49** [−.50, −.49]	−.61** [−.62, −.60]	−.59** [−.60, −.59]	
6. PANAS-C-PA	13.21	4.06	.63** [.62, .64]	.59** [.58, .59]	.64** [.64, .65]	.57** [.57, .58]	−.54** [−.55, −.53]

*Note. M* and *SD* are used to represent mean and standard deviation, respectively. Values in square brackets indicate the 95% confidence interval for each correlation. EPOCH-O = Engagement Perseverance Optimism Connectedness Happiness; SWEMWBS = Short Warwick Edinburgh Mental Well-being Scale; RSS = Rosenberg Self-Esteem Scale; M&MF-I = Me and My Feelings scale; PANAS-C-PA = PANAS-C positive affect subscale.

** *p* < .01.

## Discussion

Little attention is typically paid to fundamental structural properties of measures (Flake et al., 2017; Flake & Fried, 2020), particularly in adolescent mental health and well-being (Bentley et al., 2019; Black, Panayiotou, & Humphrey, 2022). We, therefore, sought to illustrate relevant analyses for eight mental health and well-being measures in a large sample. Conducting such analyses is crucial to avoiding bias in research (Stochl et al., 2020). In addition, brief adolescent mental health and well-being measures may need to be held to particularly strict standards where these are applied by non-researchers (for instance in schools), since models to accommodate deviation from unidimensionality or partial invariance are unfeasible in these contexts. We sought to provide evidence for a wide range of research and screening applications. Our analyses, therefore, contribute insight critical to robust use in research and have clear implications for practitioners.

Evidence in support of unidimensionality, and therefore sum scoring, was found for five measures (M&MF-I, EPOCH-O, PANAS-C-PA, SWEMWBS, and RSS). Of these five, most showed a relatively high number of non-invariant intercepts across sex and age, suggesting mean comparisons across these groups could be problematic (Steinmetz, 2013). The effect of this non-invariance on screening performance appeared less marked in general. However, M&MF-I showed substantially different sensitivity across girls and boys. No pair of measures from our range of mental health and well-being domains were correlated *r* > .70, suggesting these measures could lead to practically significant findings if used as alternatives (Carlson & Herdman, 2012). Collectively, our analyses contribute examples of the sort that might ideally be conducted more routinely in the field, specific insight into widely used measures, and demonstration of general issues such as measurement invariance.

### Unidimensionality Findings

Consistent with #BeeWell’s approach of using established measures, most showed some evidence of unidimensionality. Our results provide necessary but not sufficient evidence that the five measures meeting our criteria for unidimensionality (M&MF-I, EPOCH-O, PANAS-C-PA, SWEMWBS, and RSS) could be used for sum scoring. Although some have argued strongly that CFA should not be used to justify sum-scoring (McNeish & Wolf, 2020), others have highlighted issues with this work, including not considering the role of reliability, and false assumptions about the implications of sum-scoring (Widaman & Revelle, 2022). In addition, we drew on several methods together as others have done (Stochl et al., 2020), with particular advantages for the question of sum-scoring. First, the DFI method allowed consideration of bespoke fit consistent with no misspecification which is likely appropriate when aiming to approximate equivalence between sum and factor scores. Second, the EGA method has been shown to perform particularly well at estimating dimensionality in the presence of highly correlated subdimensions (Golino et al., 2020), meaning we provided an additional check of this scenario. Similarly, we allowed for the possibility of essential unidimensionality, which others have found useful to integrate results across psychometric models (Stochl et al., 2020). Third, our reliability results provide particular insight: For the five measures with evidence weighing in favor of unidimensionality, α and ω reliability were equivalent to the second decimal place. This is consistent with the findings of no misspecification for some of these measures (i.e., no error covariation which is an assumption of alpha; Raykov & Marcoulides, 2019), and also implies that items are all related at a similar level to the construct. Both α and ω were also high for these measures (> .81), suggesting results would be similar between observed and factor scores (Widaman & Revelle, 2022). Together these issues support sum-scoring for these five measures, and suggest risk of bias in structural models (Rhemtulla et al., 2020), or not accounting for measurement (un)reliability via structural equation modeling (Westfall & Yarkoni, 2016) may be minimal at the sample level. However, as discussed below, issues were apparent when breaking down by age and sex.

The remaining three measures (BPNSFS-A, TEIQue-ASF-ER, and PSS-4) should likely not be sum-scored or treated with caution, since clear evidence of multidimensionality was found, with at least L1 misspecification, two factors according to EGA, and failure to meet thresholds for essential unidimensionality according to bifactor indices. BPNSFS-A showed only L1 misspecification, and α and ω were relatively close and high (α = .71, ω = .74). Given this measure only has six items, it is likely reliability at the subdimension level would be undesirably lower, and that single dimension scoring might be practically better (Reise, Bonifay, et al., 2013). Given the known lack of psychometric rigor in the field in general (Bentley et al., 2019; Black, Panayiotou, & Humphrey, 2022; Flake et al., 2017), this measure may be a viable option if the specific experiences covered by the items are of particular interest. However, ideally, more work such as Rasch modeling would be conducted to validate the use of sum scores for BPNSFS-A, or alternatives should be considered or developed.

PSS-4 and TEIQue-ASF-ER showed more substantial problems according to DFI, suggesting greater challenges for treating these as unidimensional and sum-scoring. Though TEIQue-ASF-ER saw higher ω reliability, the large degree of misspecification (L3) in the model on which this is based suggests this should be disregarded.

Interestingly, each of the three measures which violated unidimensionality appeared to do so via reversed factors. For example, for the PSS-4, the two items about managing problems factored together, while the positively-framed coping items were a separate factor, consistent with other work (Demkowicz et al., 2019). It is known that reverse wording can create multidimensionality and confusion (Irwing & Hughes, 2018; van Sonderen et al., 2013), and it has been recommended that this is avoided in questionnaires with adolescents (Omrani et al., 2018). Furthermore, while reversed items may be included to account for acquiescence, the presence of a separate factor for negatively-framed items is not itself evidence of acquiescence. For instance, studies of the PSS-4 have argued the resulting factors could be interpreted as distress and coping, given the content of the items (Demkowicz et al., 2019). Therefore, if reversed items are included to assess acquiescence, this should be explicitly modeled or accounted for in some way (Kuru & Pasek, 2016; Woods, 2006). Given the highlighted need for simple approaches for the measures under study, the negatives of reverse-worded items may well outweigh the potential positives.

Considering the case in which reversed items reflect substantive rather than acquiescence, our results echo that for reliability and therefore sum scoring, reversed wording should be avoided. Indeed, practically, with such brief measures, scoring these subdimensions separately is not psychometrically robust. In addition, the level of misspecification in treating these measures as unidimensional is likely not consistent with the possibility mentioned above for the BPNSFS-A, of leveraging the total reliability, given that this was low for the total item sets. Although reliability should not always be preferenced, and broad approaches, including reverse-coding, may improve validity (Clifton, 2020), it remains unclear how this would benefit validity in adolescent mental health and well-being specifically. Work is needed to understand the impact of reverse-coding on responding and to develop the conceptualization of adolescent general mental health (Black, Panayiotou, & Humphrey, 2022). In sum, PSS-4 and TEIQue-ASF-ER are likely to pose significant problems when treated as sum scores representing single dimensions in research and school applications.

### Measurement Invariance Findings

Although reliability and structural modeling are relatively frequently included in psychometric work in adolescent mental health and well-being, consideration of measurement invariance is much rarer (Bentley et al., 2019; Black, Panayiotou, & Humphrey, 2022). Nevertheless, measurement invariance is fundamental to making valid group comparisons, which are typically sought in addition to sample-level results, particularly for age and sex. In terms of mean comparison, clear thresholds for the percentage of permissible non-invariant parameters are lacking, and this has statistical and conceptual implications (i.e., estimation and interpretability; Luong & Flake, 2021). A critical issue is that work considering the effect of partial invariance on accurate group mean recovery, often draws on *complex* models, which will not be applicable to *observed* sum score analyses (Pokropek et al., 2019). Given our aim to inform such sum score applications, we adopted the arguably strict criterion of no non-invariant intercepts consistent with work in this area by Steinmetz (2013).

All of the five unidimensional measures showed non-invariant intercepts across sex and age (25–100%), and we, therefore, suggest they are incompatible with sum score mean comparisons (Steinmetz, 2013). As indicated above, relatively little work considering the implications for sum scores is available, and we did not examine the practical effect of non-invariance on mean comparison. We are therefore somewhat cautious about recommending too strongly that such observed comparisons are abandoned, particularly given the immediate interest in the non-research applications partly motivating this paper. Nevertheless, these results suggest a need to particularly analyze and accommodate non-invariance in research, where such modeling *is* feasible to inform understanding. Although measurement invariance is relatively understudied in adolescent general mental health (Black, Panayiotou, & Humphrey, 2022), the current study suggests assuming it to hold across age and sex (i.e., not testing it), could be problematic.

In terms of screening and prevalence, when considering the 25% selection proportion across measures, sensitivity and specificity were often similar and good across sex and age, with the latter showing particularly small differences. Therefore, at the corresponding cut points (see Tables 4 & 5), selection may be relatively unbiased across sex and age despite the proportion of non-invariant intercepts, consistent with other work (Stark et al., 2004). A particular exception to this was M&MF-I for sex, where sensitivity was dramatically lower for boys (.51 compared to .94 for girls), and specificity was correspondingly lower for girls (.83 compared to .99 for boys). The cut point automatically calculated by our specification of 25% of the total sample was 9.86 (9.90 for age), which is remarkably close to the published clinical threshold of 10 (Patalay et al., 2014). Therefore, where this is applied for screening or research, it is possible boys would be missed. The fact that the most striking result was found for the internalizing symptom measure (M&MF-I) is noteworthy since it is likely this would be attractive to practitioners and researchers to estimate need (Costello, 2015; Humphrey & Wigelsworth, 2016; Soneson et al., 2020). However, our results suggest this could be the *worst* choice for that purpose among the measures in #BeeWell, particularly when considering boys.

As highlighted in recent reviews, measurement invariance analysis is typically scant in adolescent mental health and well-being (Bentley et al., 2019; Black, Panayiotou, & Humphrey, 2022), and the methods are vulnerable to bias (Crede & Harms, 2019; Kang et al., 2016; Luong & Flake, 2021). It is therefore challenging to contextualize the result for M&MF-I among other similar instruments. For instance, though we did not conduct a thorough review, studies we found considering relevant measures (internalizing, depression, and anxiety), seemed to typically report support for scalar invariance. However, these relied on, and were often close or even equal to, the more lenient CFI difference criterion of .01 (Brunet et al., 2014; Fonseca-Pedrero et al., 2012; Lu et al., 2018; Romano et al., 2021). This metric can be unreliable (Kang et al., 2016), and does not provide insight into selection bias, which some infer despite this (Brunet et al., 2014). However, in the current analysis, M&MF-I across sex did show the biggest differences for CFI, (and AIC/BIC) between configural and scalar models, and the difference for CFI of .04 exceeded even the more lenient criterion. M&MF-I may therefore show particular problems across sex (as also found in other analysis; Black et al., 2019), including for mean comparison. However, we argue it is likely not possible to determine if alternative measures are less biased, particularly for screening, given these gaps in the field.

Among the remaining selection findings, results were relatively similar between measures, with generally greater effects for sex than age, particularly for PANAS-C-PA. Of these measures, we would argue SWEMWBS may be best suited to screening or prevalence analyses since it covers a broader range of experiences than the others. Furthermore, though all items are positively phrased, several of the items relate directly to diagnostic symptom criteria (e.g., concentration and feeling relaxed; Black et al., 2021). Indeed it has been used in England’s national analysis of children and young people’s mental health (Vizard et al., 2020), and some work with adult samples has been done to link scores to depression and anxiety measures (Shah et al., 2021). More work considering the clinical validity of using SWMEWBS for prevalence or screening efforts would be needed with adolescents. Nevertheless, our findings provide tentative support for the idea that psychometric benefits of positively-framed measures could be leveraged to improve measurement accuracy with adolescents as several have suggested (Bartels et al., 2013; Black et al., 2021; Greenspoon & Saklofske, 2001; Iasiello & Agteren, 2020).

### Convergent Validity Findings

It is arguably unsurprising that the five unidimensional measures’ sum scores were not interchangeable, given that each measure could be linked to a different theoretical subdomain of well-being. However, correlations of the magnitude found here (*r* = .54–.66) are quite similar to those *within* these subdomains in other adolescent mental health convergent validity analyses, which are also often *r* < .70. For instance, Deighton et al. (2013) found the emotional symptoms subscale of the Strengths and Difficulties Questionnaire was correlated with M&MF-I at *r* = .67 in 11 to 12 year olds. Similarly, a systematic review of psychometric evidence for life satisfaction measures describes correlations *r* < .60 between similar measures (though not all explicitly life satisfaction) as evidence of validity (Proctor et al., 2009). It is therefore challenging to argue strongly that our findings provide evidence of dissociation between measures.

Our correlated but not interchangeable statistical results may be partially explained by recent work which suggests there is much common content across different domains of mental health and well-being, but that individual measures within and between theoretical domains tend not to be equivalent in terms of item content (Black, Panayiotou, & Humphrey, 2022). The current study, therefore, demonstrates the potential effects of these theoretical problems, since correlations between scores were sufficiently low as to practically affect results (Carlson & Herdman, 2012). This is important, since as described in the introduction, constructs and measures are sometimes described as if they are interchangeable, and there is a general tendency to leap from measure to construct, exaggerating the likely generalizability of a given finding (Yarkoni, 2020). However, how should similar but not interchangeable outcomes be treated in the *same* dataset? We raise this question as multiple outcomes have been recommended in adolescent mental health and well-being research (Casas & González-Carrasco, 2019; Horowitz & Garber, 2006), and it is common to collect several in observational studies (e.g., Patalay & Fitzsimons, 2018). Moreover, an entire discipline has developed out of comparing positive and negative mental health (Iasiello & Agteren, 2020).

Our findings, in light of the wider literature, suggest researchers and practitioners should carefully consider specific item content and psychometric properties relevant to their scenario. For instance, if sex comparisons are of particular interest, sex measurement invariance might be preferenced. We emphasize this because the generally underdeveloped psychometric and conceptual landscape for adolescent mental health and well-being (Bentley et al., 2019; Black, Humphrey, et al., 2022) may make it particularly vulnerable to mining for results or inferring effects (such as differences between constructs) that may be attributable to understudied measurement issues (Flake & Fried, 2020). We, therefore, argue open science practices, in which outcomes are preregistered and transparently reported are particularly needed in this field. This also suggests adolescent mental health and well-being measurement is not sufficiently developed for common measures to be recommended, as some have called for across studies (Krause et al., 2021).

### Strengths, Limitations, and Future Directions

This paper provides wide-ranging and specific insights for researchers and practitioners for key measures based on domains selected by young people in a very large dataset using comprehensive and robust analyses. Nevertheless, several limitations must be acknowledged. First, though we provided wide-ranging insight with some broad implications, findings are specific to the measures and population considered here. For instance, results are likely English-specific (Flake et al., 2017), and only a relatively narrow age range was available in the #BeeWell dataset. In addition, data were collected in autumn 2021 when the COVID-19 pandemic still greatly impacted normal life. Therefore, as with any research conducted during this time, the generalizability of the study may be affected. Similarly, we only considered self-report measures and cross-sectional data. Where researchers employ measures and analyses used here, other additional considerations may be needed, such as longitudinal invariance.

Second, we did not provide direct evidence for the sufficiency of sum scores for the five measures that showed unidimensionality, given the scope of the current paper. This could be achieved in future work via Rasch modeling or cross-validated correlations (Widaman & Revelle, 2022). Nevertheless, we integrated a range of approaches to assess unidimensionality as has been used elsewhere (Stochl et al., 2020), and robust cut-offs via DFIs. In addition, the reliability findings provided good evidence that observed sum scores are appropriate (Widaman & Revelle, 2022). Similarly, we did not directly test the effect of non-invariance on mean comparison, which should therefore also be considered in future work. Additional measurement invariance analyses beyond age and sex should also be considered across other groups such as ethnicity and special educational needs.

Third, again given the current paper’s scope, we did not explore minor modifications (e.g., removing items) to improve unidimensionality of measures. However, given the brevity of the measures, our aim to provide insight to practitioners (who are less equipped to make such adaptations), and not wanting to be too data-driven, we deemed this approach to be justified. It may be, however, that simple modifications can be applied, particularly in research contexts, to accommodate issues. Similarly, though some of the baseline models in each individual group had questionable fit, we did not make modifications (e.g., error correlations). Though this may have impacted the measurement invariance analyses, it appeared to be supported by the more acceptable fit of the configural models and is consistent with our focus on total scores.

Fourth, measurement invariance analyses and the identification of non-invariant parameters are challenging and vulnerable to a range of sample and structural issues (e.g., Kang et al., 2016). To address these issues as far as possible, we transparently reported a range of methods to judge the difference between configural and scalar models and used the automated alignment process to avoid multiple testing problems and over strictness of traditional approaches (Luong & Flake, 2021). Similarly, M&MF-I was treated as continuous in our measurement invariance analysis, as described, supported by available evidence that parameters were highly similar to DWLS results for the total sample model. Future work might consider modeling such low-category measures via an item response theory framework for selection invariance (Gonzalez & Pelham, 2020).

## Conclusion

We performed a range of robust analyses to provide insights into whether sum scoring, mean comparisons, and deployment for screening were likely to show bias for eight measures designed to assess adolescents’ mental health and well-being. Evidence for unidimensionality was found for five measures. Of these five, most showed a degree of non-invariance across sex and age likely incompatible with mean comparison. Effects on screening were less marked, except for the internalizing symptoms measure, for which sensitivity was substantially lower in boys.

Based on these findings, we argue some caution is required when applying these measures. It is also likely this caution should be extended to the broader field of adolescent mental health and well-being measures, since psychometric standards are generally low (Bentley et al., 2019; Black, Panayiotou, & Humphrey, 2022). The intended purpose of a given measure is important when considering recommendations. For example, our analyses indicate that many measures are suitable for sum scoring. However, moderate to substantial non-invariance in most of these indicates that observed score mean comparisons across sex and age—which are highly likely to be considered of interest—may be problematic. Where possible, that is, in research, measurement invariance should be examined and non-invariance explicitly modeled to better recover true mean differences. Although most measures seemed comparable and met minimal acceptability for selection purposes, M&MF-I was problematic given large differences in sensitivity between girls and boys. Ultimately, when considering the full range of our findings, and where sum scoring is the only option, SWEMWBS is likely the optimal measure among those assessed here.

Finally, we argue that the type of analyses presented here should be routinely applied by researchers to identify (and where possible, correct for) bias in adolescent mental health and well-being measures. However, our findings also highlight the need for improved development practices since those using such measures outside research contexts (e.g., schools) are unlikely to have access to models that accommodate deviation from unidimensionality or measurement invariance. Improved standards should support the “final products” that schools and other agencies use being fit for purpose. Work with adolescents is also particularly needed, and lacking (Black, Panayiotou, & Humphrey, 2022), and should focus on understanding issues uncovered here such as conceptualization of mental health and well-being, interpretation of reversed items, and potential differences between girls and boys.

## Supplemental Material

sj-docx-1-asm-10.1177_10731911231158623 – Supplemental material for Mental Health and Well-being Measures for Mean Comparison and Screening in Adolescents: An Assessment of Unidimensionality and Sex and Age Measurement Invariance Click here for additional data file. Supplemental material, sj-docx-1-asm-10.1177_10731911231158623 for Mental Health and Well-being Measures for Mean Comparison and Screening in Adolescents: An Assessment of Unidimensionality and Sex and Age Measurement Invariance by Louise Black, Neil Humphrey, Margarita Panayiotou and Jose Marquez in Assessment

## Notes

1. Alignment analyses were run from R but using Mplus since equivalent processes are not available in R.
