# Supplementary material for: Mental Health and Well-being Measures for Mean Comparison and Screening in Adolescents: An Assessment of Unidimensionality and Sex and Age Measurement Invariance
Source: Assessment. 2023 Mar 2;31(2):219–36. doi: 10.1177/10731911231158623 (PMC10822075; doi:10.1177/10731911231158623)
Supplement: sj-docx-1-asm-10.1177_10731911231158623 – Supplemental material for Mental Health and Well-being Measures for Mean Comparison and Screening in Adolescents: An Assessment of Unidimensionality and Sex and Age Measurement Invariance [file sj-docx-1-asm-10.1177_10731911231158623.docx]

Supplementary Materials for Mental Health and Wellbeing Measures for Mean Comparison and Screening in Adolescents: An Assessment of Unidimensionality and Sex and Age Measurement Invariance

Supplementary Table S1: Empirical and Dynamic Fit

|  | Empirical | L1 | L2 | L3 | Outcome | Measure |
| --- | --- | --- | --- | --- | --- | --- |
| CFI | .944 | .953 | .939 | .903 | L1 | BPNSFS-A |
| RMSEA | .097 | .089 | .107 | .144 | L1 | BPNSFS-A |
| SRMR | .054 | .053 | .057 | .063 | L1 | BPNSFS-A |
| CFI | .998 | .969 | - | - | none | EPOCH-O |
| RMSEA | .033 | .149 | - | - | none | EPOCH-O |
| SRMR | .008 | .033 | - | - | none | EPOCH-O |
| CFI | .978 | .978 | .957 | .934 | none | SWEMWBS |
| RMSEA | .064 | .065 | .094 | .119 | none | SWEMWBS |
| SRMR | .025 | .028 | .037 | .045 | none | SWEMWBS |
| CFI | .972 | .986 | .969 | - | L1 | RSS |
| RMSEA | .133 | .094 | .145 | - | L1 | RSS |
| SRMR | .027 | .019 | .029 | - | L1 | RSS |
| CFI | .742 | .911 | .865 | .845 | L3 | TEIQue-ASF-ER |
| RMSEA | .164 | .088 | .117 | .14 | L3 | TEIQue-ASF-ER |
| SRMR | .099 | .054 | .065 | .075 | L3 | TEIQue-ASF-ER |
| CFI | .909 | .983 | .948 | .915 | L3 | M&MF-I |
| RMSEA | .103 | .043 | .078 | .104 | L2 | M&MF-I |
| SRMR | .05 | .026 | .039 | .047 | L3 | M&MF-I |
| CFI | .98 | .985 | .976 | - | L1 | PANAS-C-PA |
| RMSEA | .124 | .108 | .14 | - | L1 | PANAS-C-PA |
| SRMR | .025 | .025 | .031 | - | none | PANAS-C-PA |

Supplementary Table S2 Bifactor Model Fit

|  | χ^2^ | df | *p* | CFI | RMSEA | RMSEA 95% lower CI | RMSEA 95% upper CI | SRMR |
| --- | --- | --- | --- | --- | --- | --- | --- | --- |
| BPNSFS-A | 49.435 | 5 | <.001 | .999 | .016 | .012 | .021 | .005 |
| TEIQue-ASF-ER | 461.96 | 4 | <.001 | .985 | .059 | .055 | .064 | .023 |
| PSS | 418.191 | 1 | <.001 | .983 | .112 | .103 | .121 | .03 |

Supplementary Table S3 Baseline Models

| χ^2^ | df | *p* | CFI | RMSEA | SRMR | AIC | BIC | Group | Measure | *N* | DFI results |
| --- | --- | --- | --- | --- | --- | --- | --- | --- | --- | --- | --- |
| 51.723 | 2 | <.001 | .998 | .039 | .01 | 187027.897 | 187089.546 | F | EPOCH-O | 18658 | none_none_none |
| 23.1 | 2 | <.001 | .999 | .025 | .007 | 189130.619 | 189192.34 | M | EPOCH-O | 19289 | none_none_none |
| 38.034 | 2 | <.001 | .998 | .032 | .009 | 201180.854 | 201242.972 | 8 | EPOCH-O | 20383 | none_none_none |
| 34.671 | 2 | <.001 | .999 | .032 | .008 | 176229.374 | 176290.611 | 10 | EPOCH-O | 17595 | none_none_none |
| 931.046 | 14 | <.001 | .978 | .064 | .025 | 306292.676 | 306400.389 | F | SWEMWBS | 18658 | none_none_none |
| 921.793 | 14 | <.001 | .977 | .063 | .025 | 308189.843 | 308297.71 | M | SWEMWBS | 19289 | none_none_none |
| 775.942 | 14 | <.001 | .982 | .056 | .023 | 329978.194 | 330086.73 | 8 | SWEMWBS | 20383 | none_none_none |
| 1226.745 | 14 | <.001 | .972 | .075 | .028 | 287210.822 | 287317.843 | 10 | SWEMWBS | 17595 | L1_L1_L1 |
| 1574.822 | 5 | <.001 | .969 | .138 | .029 | 149259.764 | 149336.83 | F | RSS | 18658 | L1_L1_L1 |
| 1217.04 | 5 | <.001 | .976 | .12 | .025 | 145356.737 | 145434.01 | M | RSS | 19289 | L1_L1_L1 |
| 1216.419 | 5 | <.001 | .978 | .117 | .024 | 157777.401 | 157855.145 | 8 | RSS | 20383 | L1_L1_L1 |
| 1756.018 | 5 | <.001 | .965 | .15 | .03 | 139642.45 | 139719.028 | 10 | RSS | 17595 | L2_L2_L3 |
| 6695.119 | 35 | <.001 | .895 | .107 | .052 | 285470.067 | 285624.312 | F | M&MF-I | 18658 | L3_L3_L3 |
| 5528.047 | 35 | <.001 | .906 | .097 | .05 | 264031.472 | 264186.065 | M | M&MF-I | 19289 | L2_L2_L2 |
| 615.585 | 35 | <.001 | .912 | .099 | .048 | 295729.216 | 295884.874 | 8 | M&MF-I | 20383 | L2_L2_L2 |
| 637.952 | 35 | <.001 | .905 | .108 | .052 | 264322.919 | 264476.046 | 10 | M&MF-I | 17595 | L3_L3_L3 |
| 1373.106 | 5 | <.001 | .978 | .127 | .028 | 205634.371 | 205711.721 | F | PANAS_C_PA | 18658 | L1_L1_L1 |
| 1222.549 | 5 | <.001 | .982 | .119 | .024 | 19676.119 | 196837.602 | M | PANAS_C_PA | 19289 | L1_L1_none |
| 1408.022 | 5 | <.001 | .979 | .125 | .026 | 217745.313 | 217823.341 | 8 | PANAS_C_PA | 20383 | L1_L1_L1 |
| 1218.624 | 5 | <.001 | .981 | .123 | .025 | 186275.346 | 186352.129 | 10 | PANAS_C_PA | 17595 | L1_L1_none |

Supplementary Table S4 configural/scalar models

| Model | χ^2^ | df | *p* | CFI | RMSEA | SRMR | AIC | BIC | Δ χ^2^ *p* | Δ CFI | Δ AIC | Δ BIC |
| --- | --- | --- | --- | --- | --- | --- | --- | --- | --- | --- | --- | --- |
| Sex configural  EPOCH-O | 74.823 | 4 | < .001 | .998 | .033 | .007 | 376174.516 | 376376.207 | - | - | - | - |
| Sex scalar  EPOCH-O | 195.891 | 10 | < .001 | .996 | .034 | .018 | 376283.584 | 376434.852 | < .001 | .003 | -109.068 | -58.645 |
| Age configural  EPOCH-O | 72.705 | 4 | < .001 | .998 | .032 | .007 | 377426.227 | 377627.933 | - | - | - | - |
| Age scalar  EPOCH-O | 118.514 | 10 | < .001 | .997 | .026 | .011 | 37746.036 | 377611.316 | < .001 | .001 | -33.809 | 16.617 |
| Sex configural  SWEMWBS | 1852.839 | 28 | < .001 | .977 | .063 | .023 | 61451.519 | 614863.003 | - | - | - | - |
| Sex scalar  SWEMWBS | 2542.347 | 40 | < .001 | .969 | .062 | .033 | 615176.027 | 615427.801 | < .001 | .008 | -665.508 | -564.799 |
| Age configural  SWEMWBS | 2002.686 | 28 | < .001 | .977 | .066 | .023 | 617217.016 | 617569.525 | - | - | - | - |
| Age scalar  SWEMWBS | 2150.642 | 40 | < .001 | .975 | .057 | .025 | 61734.972 | 617592.764 | < .001 | .002 | -123.956 | -23.239 |
| Sex configural  RSS | 2791.862 | 10 | < .001 | .973 | .129 | .023 | 294636.501 | 294888.806 | - | - | - | - |
| Sex scalar  RSS | 3819.26 | 18 | < .001 | .963 | .113 | .039 | 295647.899 | 295832.922 | < .001 | .01 | -1011.398 | -944.117 |
| Age configural  RSS | 2972.436 | 10 | < .001 | .972 | .134 | .023 | 297439.851 | 297692.179 | - | - | - | - |
| Age scalar  RSS | 3044.233 | 18 | < .001 | .971 | .101 | .025 | 297495.647 | 29768.687 | < .001 | .001 | -55.796 | 11.491 |
| Sex configural  M&MF-I | 12223.166 | 70 | < .001 | .900 | .102 | .047 | 549541.539 | 550046.386 | - | - | - | - |
| Sex scalar  M&MF-I | 16473.786 | 88 | < .001 | .865 | .106 | .072 | 553756.159 | 554109.552 | < .001 | .035 | -4214.62 | -4063.166 |
| Age configural  M&MF-I | 12521.538 | 70 | < .001 | .909 | .103 | .046 | 560092.135 | 560597.022 | - | - | - | - |
| Age scalar  M&MF-I | 12872.919 | 88 | < .001 | .906 | .093 | .048 | 560407.516 | 56076.937 | < .001 | .002 | -315.381 | -163.915 |
| Sex configural  PANAS-C-PA | 2595.655 | 10 | < .001 | .980 | .123 | .022 | 402414.489 | 402667.535 | - | - | - | - |
| Sex scalar  PANAS-C-PA | 3184.061 | 18 | < .001 | .975 | .102 | .032 | 402986.895 | 403172.462 | < .001 | .005 | -572.406 | -504.927 |
| Age configural  PANAS-C-PA | 2626.646 | 10 | < .001 | .980 | .124 | .022 | 40404.66 | 404293.728 | - | - | - | - |
| Age scalar  PANAS-C-PA | 2731.536 | 18 | < .001 | .979 | .094 | .024 | 404129.55 | 404315.133 | < .001 | .001 | -88.89 | -21.405 |

*Note.* CFI/AIC/BIC difference is calculated as the configural model value minus the scalar model value.


Supplementary Table S5 Non-invariant Items Identified After Alignment Analysis and used in Partially-Invariant Models

|  | Sex | | Age | |
| --- | --- | --- | --- | --- |
| Measure | Loadings | Intercepts | Loadings | Intercepts |
| EPOCH-O | 2: In uncertain times, I expect the best.  4: I believe that things will work out, no matter how difficult they seem. | 4: I believe that things will work out, no matter how difficult they seem. | - | 4: I believe that things will work out, no matter how difficult they seem. |
| SWEMWBS | 1: I’ve been feeling optimistic about the future  2: I’ve been feeling useful.  4: I’ve been dealing with problems well.  5: I’ve been thinking clearly.  7: I’ve been able to make up my own mind about things. | 1: I’ve been feeling optimistic about the future  2: I’ve been feeling useful.  3: I’ve been feeling relaxed.  4: I’ve been dealing with problems well.  5: I’ve been thinking clearly.  6: I’ve been feeling close to other people. | 5: I’ve been thinking clearly. | 1: I’ve been feeling optimistic about the future  3: I’ve been feeling relaxed.  4: I’ve been dealing with problems well.  5: I’ve been thinking clearly.  6: I’ve been feeling close to other people. |
| RSS | 1: On the whole, I am satisfied with myself.  2: I feel that I have a number of good qualities.  3: I am able to do things as well as most other people. | 1: On the whole, I am satisfied with myself.  3: I am able to do things as well as most other people.  4: I am a person of value.  5: I feel good about myself. | - | 1: On the whole, I am satisfied with myself.  4: I am a person of value.  5: I feel good about myself. |
| M&MF-I | 1: I feel lonely  2: I am unhappy  4: I cry a lot.  5: I worry when I am at school.  9: I am shy. | 1: I feel lonely  2: I am unhappy  3: Nobody likes me  4: I cry a lot.  5: I worry when I am at school.  6: I worry a lot.  7: I have problems sleeping  8: I wake up in the night.  9: I am shy.  10: I feel scared | 1: I feel lonely  2: I am unhappy  7: I have problems sleeping  8: I wake up in the night. | 1: I feel lonely  2: I am unhappy  4: I cry a lot.  6: I worry a lot.  8: I wake up in the night.  9: I am shy.  10: I feel scared |
| PANAS-C-PA | 2: Cheerful.  5: Proud. | 1: Joyful  2: Cheerful.  3: Happy.  4: Lively.  5: Proud. | 5: Proud. | 1: Joyful  2: Cheerful.  5: Proud. |

Supplementary Table S6 Fit of Partial Invariance Models

| Model | χ^2^ | df | *p* | CFI | AIC | BIC | RMSEA | RMSEA 95% lower CI | RMSEA 95% upper CI | SRMR |
| --- | --- | --- | --- | --- | --- | --- | --- | --- | --- | --- |
| EPOCH-O sex | 83.797 | 7 | <.001 | .998 | 376177.49 | 376353.97 | .026 | .021 | .031 | .008 |
| EPOCH-O age | 92.738 | 9 | <.001 | .998 | 377436.26 | 377595.944 | .024 | .02 | .028 | .01 |
| SWEMWBS sex | 1856.883 | 29 | <.001 | .977 | 614512.563 | 614856.654 | .062 | .06 | .065 | .023 |
| SWEMWBS age | 2016.323 | 34 | <.001 | .977 | 617218.652 | 61752.803 | .06 | .058 | .062 | .023 |
| RSS sex | 2791.862 | 10 | <.001 | .973 | 294636.501 | 294888.806 | .129 | .125 | .134 | .023 |
| RSS age | 2983.698 | 15 | <.001 | .972 | 297441.113 | 297651.385 | .109 | .106 | .112 | .024 |
| PANAS-C-PA  sex | 2601.298 | 12 | <.001 | .98 | 402416.133 | 402652.309 | .113 | .109 | .116 | .022 |
| PANAS-C-PA  age | 2632.723 | 14 | <.001 | .98 | 404038.737 | 404258.063 | .105 | .101 | .108 | .022 |
| M&MF-I sex | 12239.038 | 74 | <.001 | .9 | 549549.411 | 550020.602 | .099 | .098 | .101 | .047 |
| M&MF-I age | 12548.98 | 77 | <.001 | .909 | 560105.578 | 560551.561 | .099 | .097 | .1 | .046 |
